# Novel complete mitochondrial genomes of eight riverine *Lamprologus* species (Actinopterygii, Cichlidae) suggest in-situ speciation of the blind cichlid *L. lethops* in the lower Congo River

**DOI:** 10.1080/23802359.2025.2519212

**Published:** 2025-06-17

**Authors:** Sebastian M. Jimenez, Naoko P. Kurata, Melanie L. J. Stiassny, S. Elizabeth Alter, Prosanta Chakrabarty, Fernando Alda

**Affiliations:** a Department of Biology, Geology and Environmental Science, University of Tennessee at Chattanooga, Chattanooga, Tennessee, USA; b The Graduate Center of the City University of New York, New York, New York, USA; c Department of Ichthyology, American Museum of Natural History, New York, New York, USA; d The Sackler Institute for Comparative Genomics, American Museum of Natural History, New York, New York, USA; e Department of Biology and Chemistry, California State University Monterey Bay, Seaside, California, USA; f Museum of Natural Science and Department of Biological Science, Louisiana State University, Baton Rouge, Louisiana, USA; g SimCenter: Center for Excellence in Applied Computational Science and Engineering, University of Tennessee at Chattanooga, Chattanooga, Tennessee, USA; h Instituto de Investigación en Recursos Cinegéticos (IREC; CSIC-UCLM-JCCM), Ciudad Real, Spain

**Keywords:** Cichlids, Congo River, introgression, lamprologines, mitogenomes, phylogeny

## Abstract

Lamprologine cichlids include nearly 100 species from Lake Tanganyika, but only nine are known from the Congo River, including *Lamprologus lethops*, the only known blind cichlid. Little is known about its natural history. We characterized the complete mitochondrial genomes of *L. lethops* and seven related riverine species to infer evolutionary relationships. Genomes were similar in size and structure. Riverine Lamprologus formed two non-sister mitochondrial lineages more closely related to Lake Tanganyika lamprologines than to each other, suggesting past introgression or incomplete lineage sorting. *Lamprologus lethops* was sister to lower Congo River species. Broader taxonomic and genomic sampling is needed.

## 1. Introduction

**Figure 1.**
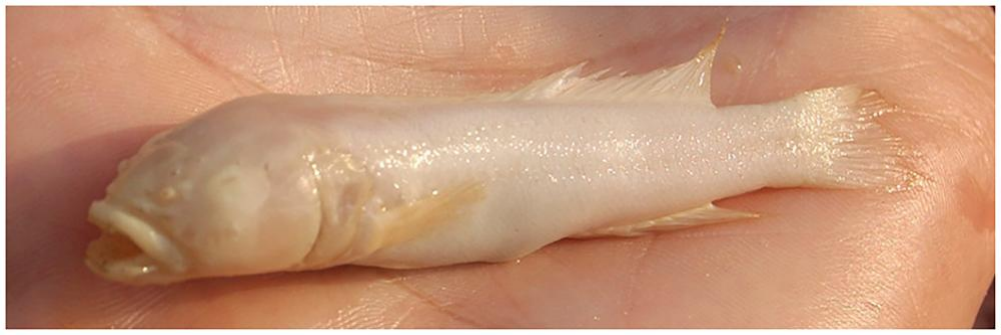
Specimen of *Lamprologus lethops* (c. 70 mm long) on a hand. Photograph by the American Museum of Natural History, used with permission and courtesy of Melanie L. J. Stiassny.

Lamprologine cichlids are a fascinating group of fish that display remarkable diversity in their morphology, behavior, and ecology (Stiassny 1997). As currently recognized, the genus *Lamprologus* comprises 20 species within the tribe Lamprologini that are mainly found in Lake Tanganyika and associated streams but unlike other genera within the lamprologines, *Lamprologus* also includes fully riverine species found throughout the Congo River drainage (Schelly and Stiassny 2004; Ronco et al. 2020; Stiassny and Alter 2021). The riverine species of *Lamprologus* occupy distinct and mostly non-overlapping ranges along the Congo River. Compared to their lacustrine congeners, they exhibit some unique adaptations to the often extreme environmental conditions they encounter, including fluctuating water levels and strong currents (Stiassny and Alter 2021; Kurata et al. 2022). Without question, the most remarkable species in the lower Congo River (LCR) is *Lamprologus lethops* Roberts and Stewart 1976, the only known cryptophthalmic and fully depigmented cichlid species (Figure 1). *Lamprologus lethops* is morphologically strikingly divergent from its riverine congeners, and it is presumed to inhabit the deepest portions of the LCR, where it is only known from specimens that are occasionally found at the surface dead or moribund (Schobert et al. 2013; Stiassny and Alter 2021; Kurata et al. 2022).

The extreme environments of the LCR have been hypothesized to facilitate the generation and maintenance of species divergence and isolation (Markert et al. 2010; Alter et al. 2015; Kurata et al. 2024). However, the patterns of diversification and relationships among species remain poorly understood. Recent genome-wide phylogenetic analyses support the monophyly of riverine *Lamprologus* species. However, high levels of gene tree discordance prevent a confident resolution of relationships within this clade (Astudillo-Clavijo et al. 2023; Alda et al. 2025). Mitochondrial DNA, with its smaller effective population size and faster mutation rate, provides a complementary approach for phylogenetic studies by mitigating the effects of incomplete lineage sorting and capturing phylogenetic signals from rapid divergence (Avise and Ellis 1986; Harrison 1989), as observed in cichlid radiations. Despite this potential, mitochondrial-based studies have consistently failed to recover both the genus *Lamprologus* and the riverine clade as monophyletic. However, these studies have so far been limited by taxonomically incomplete sampling or reliance on a small number of loci (Day et al. 2007; Schedel et al. 2019).

In the current study, we aim to contribute to the resolution of these knowledge gaps by describing the mitochondrial DNA diversity of *Lamprologus* using the first complete mitochondrial genomes of eight riverine species including the blind cichlid *L. lethops* and inferring their evolutionary relationships.

## 2. Materials and methods

### 2.1. Specimens studied

We analyzed 11 samples of eight species of *Lamprologus* that inhabit the Congo River: *Lamprologus congoensis* Schilthuis 1891 (AMNH 257860, AMNH255211)*, Lamprologus lethops* Roberts and Stewart 1976 (AMNH263957)*, Lamprologus markerti* Tougas and Stiassny 2014 (AMNH238650)*, Lamprologus mocquardi* Pellegrin 1903 (ZSM Kis-2008-080, ZSM Kis-2008-003)*, Lamprologus teugelsi* Schelly and Stiassny 2004 (AMNH238649)*, Lamprologus tigripictilis* Schelly and Stiassny 2004 (AMNH263989)*, Lamprologus tumbanus* Boulenger 1898 (AMNH247886), and *Lamprologus werneri* Poll 1959 (AMNH239703, AMNH263499) (Table 1). We also analyzed two non-*Lamprologus* species: *Julidochromis dickfeldi* Staeck, 1975 (AMNH26257) and *Telmatochromis burgeoni* Poll 1942 (AMNH264405), and sequences from available lamprologine partial mitogenomes (Schedel et al. 2019), that were used as outgroups (Table 1). All fishes, except *L. lethops* which was recovered moribund at the river surface near Bulu (Aardema et al. 2020), were collected alive and euthanized using an overdose of MS-222 (250 mg/L) in compliance with ethical and legal guidelines for international animal research approved by the AMNH Institutional Animal Care and Use Committee (IACUC) (approval #36/06). Voucher specimens and tissues are deposited in the Ichthyology collections of the American Museum of Natural History (https://nhm.org/research-collections/departments-and-programs/ichthyology, curator: Melanie L. J. Stiassny, mljs@amnh.org) and the Zoologische Staatssammlung München (Bavarian State Collection of Zoology; https://zsm.snsb.de/sektion/ichthyology/?lang=en, curator: Ulrich Schliewen, Schliewen@snsb.de) (Location and voucher information are provided in Table 1).

**Table 1.** List of samples analyzed in this study.

Species	Sample code	Voucher	Locality	Latitude	Longitude	Genbank acc. no.	Source
*Lamprologus congoensis*	T8	AMNH 257860	N’Dolo Pool Malebo, Dem. Rep. Congo (2011)	−4.29	15.33	OQ862828	This study (UCE)
*Lamprologus congoensis*	T12	AMNH 255211	Main channel of middle Congo River at rocky outcrop above guard post at Nkana, Dem. Rep. Congo (2011)	N/A	N/A	OQ817999	This study (UCE)
*Lamprologus lethops*		AMNH 263957	Unknown locality in Bulu, Dem. Rep. Congo (2014)	N/A	N/A	OR286605	Aardema et al. 2020 (WGS)
*Lamprologus markerti*	E30	AMNH 238650	Nziya, downstream of Inga below Bundi stream at Congo River confluence, Dem. Rep. Congo (2005)	−5.55	13.55	OQ818000	This study (UCE)
*Lamprologus mocquardi*	E124	ZSM-KIS-2008-080	Nki, Cameroon (2008)	N/A	N/A	OQ862829	This study (UCE)
*Lamprologus mocquardi*	E125	ZSM-KIS-2008-003	Kisangani, Dem. Rep. Congo (2008)	N/A	N/A	OQ862830	This study (UCE)
*Lamprologus teugelsi*	E41	AMNH 238649	Nziya, downstream of Inga below Bundi stream at Congo River confluence, Dem. Rep. Congo (2005)	−5.55	13.55	OQ862831	This study (UCE)
*Lamprologus tigripictilis*		AMNH 263989	Vicinity of Luozi, Dem. Rep. Congo (2015)	−4.93	14.18	OQ862832	Aardema et al. 2020 (WGS)
*Lamprologus tumbanus*	E61	AMNH 247886	Lake Tumba, Dem. Rep. Congo (2008)	−0.73	18.13	OQ862833	This study (UCE)
*Lamprologus werneri*	E116	AMNH 239703	Les Rapides, Congo (2006)	−4.31	15.23	OQ862834	This study (UCE)
*Lamprologus werneri*	T17	AMNH 263499	Mbudi, Dem. Rep. Congo (2014)	−4.37	15.18	OQ862835	This study (UCE)
*Telmatochromis burgeoni*	E75	AMNH 264405	Onzye Point, Lake Tanganyika	−8.75	31.10	OQ862837	This study (UCE)
*Julidochromis dickfeldi*	E06	AMNH 265257	Aquarium specimen, Lake Tanganyika			OQ862836	This study (UCE)
*Altolamprologus calvus*		ZSM-PIS-040877	Aquarium specimen, Lake Tanganyika			MK144669	Schedel et al. 2019
*Chalinochromis* sp.		ZSM-PIS-040878	Aquarium specimen, Lake Tanganyika			MK144684	Schedel et al. 2019
*Lamprologus* cf*. teugelsi*		ZSM-PIS-042643	Congo River, Dem. Rep. Congo			MK144718	Schedel et al. 2019
*Lamprologus lethops*		ZSM-PIS-038320	Congo River, Dem. Rep. Congo			MK144719	Schedel et al. 2019
*Lamprologus markerti*		ZSM-PIS-042658	Congo River, Dem. Rep. Congo			MK144720	Schedel et al. 2019
*Lamprologus mocquardi*		ZSM-PIS-037545	Tshopo River, Dem. Rep. Congo			MK144721	Schedel et al. 2019
*Lamprologus signatus*		ZSM-PIS-040803	Aquarium specimen, Lake Tanganyika			MK144722	Schedel et al. 2019
*Lamprologus* sp.		ZSM-PIS-038830	Kwango River, Dem. Rep. Congo			MK144723	Schedel et al. 2019
*Lamprologus symnoensi*		–	Lovoi River, Dem. Rep. Congo			MK144724	Schedel et al. 2019
*Lamprologus tigripictilis*		ZSM-PIS-042661	Congo River, Dem. Rep. Congo			MK144725	Schedel et al. 2019
*Lamprologus werneri*		ZSM-PIS-037841	Congo River, Dem. Rep. Congo			MK144726	Schedel et al. 2019
*Lepidiolamprologus nkambae*		ZSM-PIS-040795	Aquarium specimen, Lake Tanganyika			MK144727	Schedel et al. 2019
*Neolamprologus brichardi*		ZSM-PIS-040802	Aquarium specimen, Lake Tanganyika			MK144733	Schedel et al. 2019
*Telmatochromis* cf. *temporalis*		ZSM-PIS-040829	Aquarium specimen, Lake Tanganyika			MK144770	Schedel et al. 2019
*Telmatochromis* sp.		–	Aquarium specimen, Lufubu River, Zambia			MK144771	Schedel et al. 2019
*Variabilichromis moorii*		ZSM-PIS-040832	Aquarium specimen, Lake Tanganyika			MK144784, MK144785	Schedel et al. 2019
*Neolamprologus brichardi*		–	–			NC_009062	Mabuchi et al. 2007

AMNH: American Museum of Natural History, ZSM: Zoologische Staatssammlung München (Bavarian State Collection of Zoology), UCE: ultraconserved elements, WGS: whole genome sequencing.

### 2.2. Mitogenome sequencing, assembly, and annotation

We obtained mitochondrial sequences as a by-product of genomic libraries enriched for 500 ultraconserved elements (UCEs) across Actinopterygii, using a set of 2001 probes (Actinopts-UCE-0.5Kv1; Faircloth et al. 2013), following methods outlined at http://ultraconserved.org with slight modifications (PRJNA1097814; Burress et al. 2018; Alda et al. 2025). Additional mitochondrial sequences were retrieved from whole genome libraries (Aardema et al. 2020, BioProject PRJNA577474) (Table 1). We used Geneious Prime 2022.1.1 (https://www.geneious.com) to trim our raw reads for low-quality bases (cut-off limit: 0.05) and map them to the closest reference mitochondrial genome available, which we used as reference (*Neolamprologus brichardi*, NC_009062, Mabuchi et al. 2007). We then created consensus sequences and transferred the annotations from our reference genome to each sample.

### 2.3. Phylogenetic analysis

For the phylogenetic analysis, we used protein-coding genes extracted from our complete mitochondrial genomes, aligning them alongside available partial genomes using MAFFT 1.5.0 (Katoh and Standley 2013). The protein-coding gene sequences were concatenated in a single alignment, partitioned by gene and codon. We estimated the best arrangement of partitions and their nucleotide substitution model using Partition Finder (Lanfear et al. 2017) in IQ-TREE2 (Minh et al. 2013). We inferred a maximum-likelihood tree from the protein-coding gene alignment and assessed nodal support using 1000 ultrafast bootstrap replicates (Hoang et al. 2018). Finally, we used the approximately unbiased (AU) test (Shimodaira 2002) (α = 0.05) to test the monophyly of the riverine *Lamprologus* by comparing an unconstrained maximum-likelihood tree and a constrained tree in which riverine species were forced to form a monophyletic group.

## 3. Results

### 3.1. Characteristics of *Lamprologus* mitogenomes

**Figure 2.**
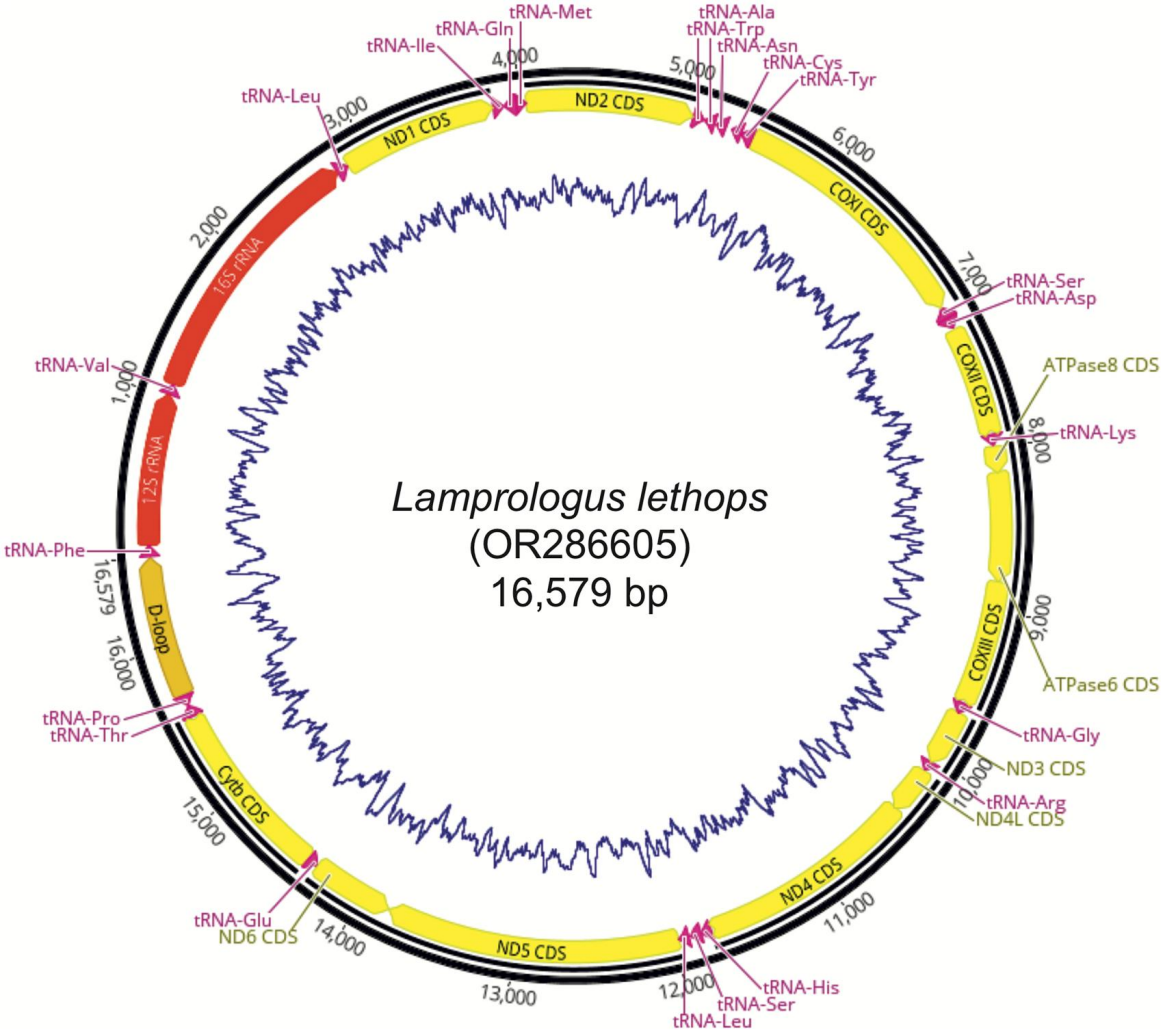
Graphical representation of the complete mitochondrial genome of *Lamprologus lethops* showing the annotation of all protein-coding genes (yellow arrows), rRNA genes (red arrows), tRNA genes (pink arrows), and control region (tan arrow). The blue line represents GC content. For color codes, refer to the online version of this article.

We recovered complete genomes from all individuals except for *L. markerti,* which was missing 33 bp in the *ND3* gene and 93 bp in the *ND5* gene, and *L. teugelsi* which was missing 174 bp in the *ND1* gene. The mean coverage of the genomes recovered from UCE raw sequence data was 57.96✕ ±18.78 SD, and 3872✕ ±702.10 SD for those recovered from whole genome sequencing data (Fig. S1). All species genomes consisted of 22 tRNA genes, two rRNA genes, 13 protein-coding genes, and a control region (D-loop) in identical order (Table S1). The mean base composition was A: 26.95% ±0.121 SD; C: 30.94% ±2.27 SD; G: 16.31% ±0.19 SD; and T: 26.67% ±0.205 SD. The genome lengths ranged from 16,579 bp in *L. lethops* to 16,587 bp in *L. mocquardi* (Figure 2).

The most common start codon was ATG (Met), except for GTG (Val) that was the start codon in the *COX1* gene in all species. The most common stop codon was TAA, followed by T–. In *ND1*, TAG was the stop codon in all species except in *L. teugelsi*, which used TAA. The stop codon of *ND2* was T– in *L. lethops*, *L. markerti*, *L. teugelsi*, and *L. tigripictilis*, and TA- in *L. congoensis*, *L. mocquardi*, *L. tumbanus*, and *L. werneri* (Table S1).

### 3.2. Mitochondrial phylogeny of riverine *Lamprologus*

**Figure 3.**
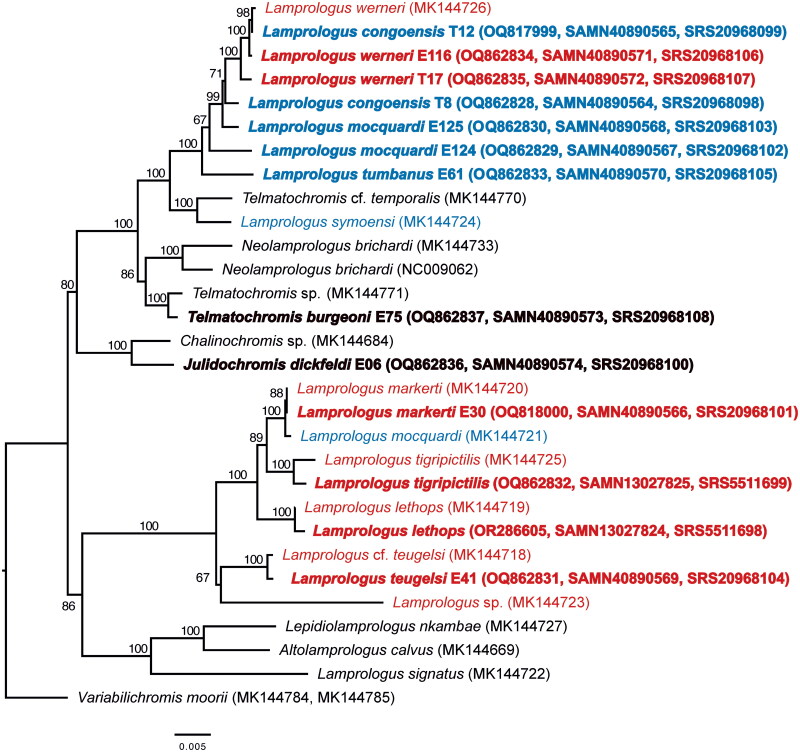
Maximum-likelihood (IQ-TREE 2) phylogeny based on all mitochondrial protein-coding genes of riverine *Lamprologus* species analyzed in this study (in bold) along with additional lamprologine species with available partial mitogenomes (Mabuchi et al. 2007; Schedel et al. 2019). Species endemic to the Lower Congo River are marked in red, and those endemic to the Middle and Upper Congo River are shown in blue. Node labels indicate support values obtained after 1000 ultrafast bootstrap replicates, and GenBank accession numbers are between parentheses. Sample details are indicated in Table 1.

The best maximum-likelihood tree recovered species of riverine *Lamprologus* in two lineages (Figure 3). The first lineage included a clade composed exclusively of riverine *Lamprologus* (*L. werneri*, *L. congoensis*, *L. mocquardi*, *L. tumbanus*). The second lineage also included a clade of riverine *Lamprologus*, in which *L. markerti* and *L. mocquardi* were closely related and sister to *L. tigripictilis*, with *L. lethops* as sister to them. *Lamprologus teugelsi* and *L.* sp. from the Kwango River also belonged to this clade (Figure 3).

## 4. Discussion

Mitogenomes of riverine *Lamprologus* were identical in the number and order of genes and similar in size to other lamprologines (Mabuchi et al. 2007). We recovered the riverine *Lamprologus* in two non-sister mitochondrial lineages, in which individuals of the same species (*L. congoensis*, *L. mocquardi*, *L. werneri*) were not always resolved as one another’s closest relatives.

Genetic sequence data from the enigmatic *L. lethops* are scarce and this study provides the first complete mitochondrial genome for the species (Figure 2). Similar to a recent genomic study, mitogenomic data recovered *L. lethops* in a clade comprising LCR species such as *L. tigripictilis*, *L. markerti*, *L. werneri* and *L. teugelsi* (Astudillo-Clavijo et al. 2023). Despite the differences, all studies suggest that *L. lethops* originated through a process of *in situ* speciation within the LCR.

Schedel et al. (2019) recovered a clade consisting solely of LCR species. However, with the inclusion of additional specimens in our analysis, the monophyly of the LCR group was statistically rejected (p-value of AU test = 1.32 × 10^−7^). This result contrasts with both morphological and phylogenomic hypotheses that support the monophyly of riverine *Lamprologus* (Schelly and Stiassny 2004; Schelly 2006), including studies that have analyzed several of the same specimens examined in this work (Astudillo-Clavijo et al. 2023; Alda et al. 2025). In particular, the phylogenomic analysis of ultraconserved elements conducted on the same individuals from which mitochondrial genomes were extracted recovered reciprocally monophyletic *Lamprologus* lineages from the Lower Congo and the Middle and Upper Congo river regions, which were inferred to be sister groups (Fig. S2). Mito-nuclear discordance and even complete mitochondrial replacements are not uncommon among syntopic lamprologines in Lake Tanganyika (Schelly 2006; Koblmüller et al. 2007; Nevado et al. 2009). These patterns are often attributed to incomplete lineage sorting (ILS) and both past and ongoing introgressive hybridization. In contrast, *Lamprologus* species in the Congo River show allopatric or parapatric distributions, with little gene flow inferred between neighboring species (Kurata et al. 2022), along with geographical structure of mitochondrial haplotypes. Thus, ancient hybridization events, rather than ongoing gene flow or random ILS, are the more likely explanation for the observed phylogenetic discordance. However, other processes, such as ILS, ongoing hybridization, or even selection against specific lineages, cannot be ruled out based on the available data.

## 5. Conclusions

Overall, our study uncovered previously undescribed mitochondrial lineages and diversity among riverine *Lamprologus*, offering valuable insights into the evolutionary history of this group. The rejection of LCR monophyly, along with the identification of two non-sister mitochondrial lineages and evidence of phylogenetic discordance, underscores the intricate genetic relationships within these species. These findings highlight the need to analyze larger sample sizes and the integration of nuclear and mitochondrial genomic data to better understand the processes driving reticulated evolution of *Lamprologus* and other fish groups in biodiversity hotspots like the Congo River system.

## Supplementary Material

Jimenezetal_mtDNAPartB_MitogenomeReport_SupplMat_R3.pdf

## Data Availability

The raw sequence data that support the findings of this study are openly available in GenBank of NCBI at https://www.ncbi.nlm.nih.gov/ under BioProjects PRJNA577474 (BioSample numbers: SAMN13027824–SAMN13027825, SRA numbers: SRX6989555–SRX6989556) and PRJNA1097814 (BioSample numbers: SAMN40890564–SAMN40890574, and SRA numbers: SRX24194295–SRX24194305). The accession numbers of the assembled mitochondrial genomes are: OQ817999, OQ818000, OQ862828, OQ862829, OQ862830, OQ862831, OQ862832, OQ862833, OQ862834, OQ862835, OQ862836, OQ862837 and OR286605 (see Table 1 for details). Additional supporting data for this study (sequence alignments and annotated genomes as General Feature Format files) are openly available in Zenodo at https://doi.org/10.5281/zenodo.11442789. A preprint of this article is deposited in bioRxiv at https://doi.org/10.1101/2024.09.11.612419 (Jimenez et al. 2024).
