# Supplementary material for: Novel complete mitochondrial genomes of eight riverine Lamprologus species (Actinopterygii, Cichlidae) suggest in-situ speciation of the blind cichlid L. lethops in the lower Congo River
Source: Mitochondrial DNA B Resour. 2025 Jun 17;10(7):595–601. doi: 10.1080/23802359.2025.2519212 (PMC12175219; doi:10.1080/23802359.2025.2519212)
Supplement: Jimenezetal_mtDNAPartB_MitogenomeReport_SupplMat_R3.pdf [file TMDN_A_2519212_SM5687.pdf]

Supplementary Material

Novel complete mitochondrial genomes of eight riverine *Lamprologus* species (Actinopterygii, Cichlidae) suggest in-situ speciation of the blind cichlid *L. lethops* in the lower Congo River

Sebastian M. Jimenez, Naoko P. Kurata, Melanie L. J. Stiassny, S. Elizabeth Alter, Prosanta Chakrabarty, Fernando Alda

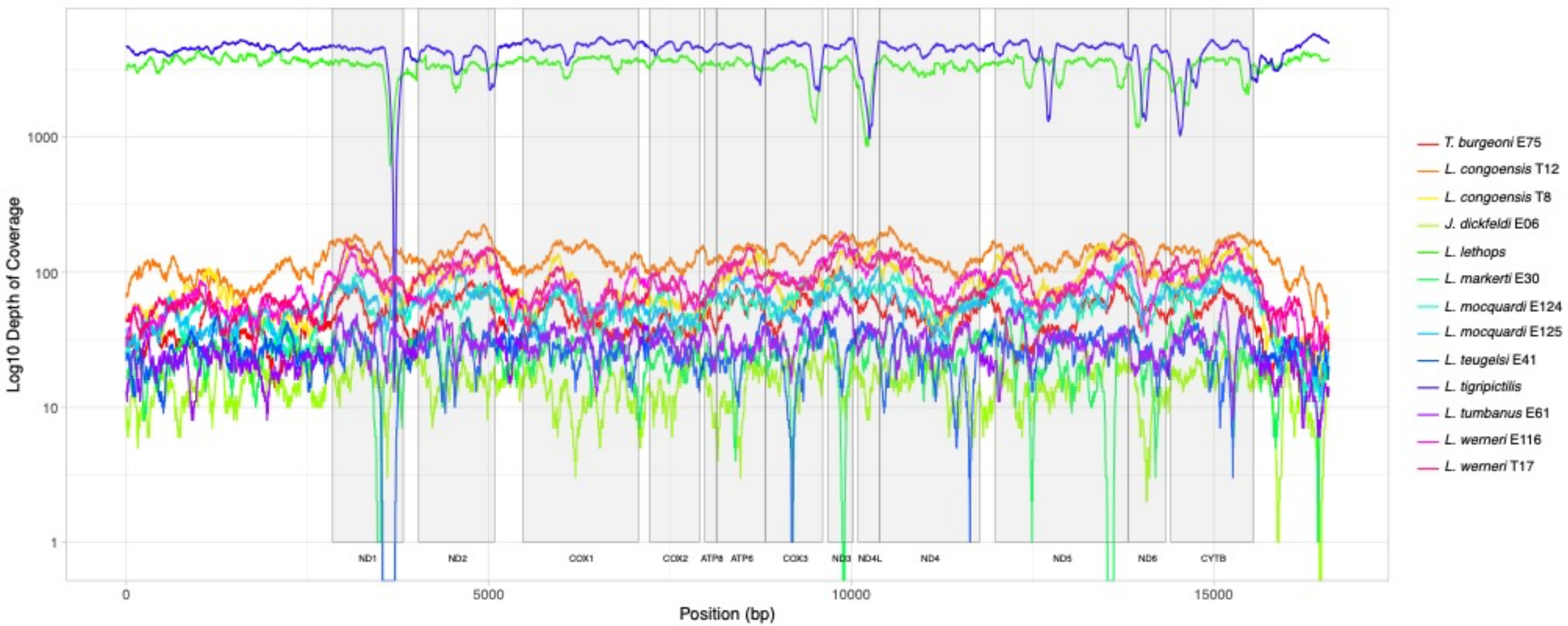

Fig. S1 Read depth of coverage plot of all the mitochondrial genomes assembled in this study. For reference, grey boxes indicate the location of the mitochondrial protein coding genes.

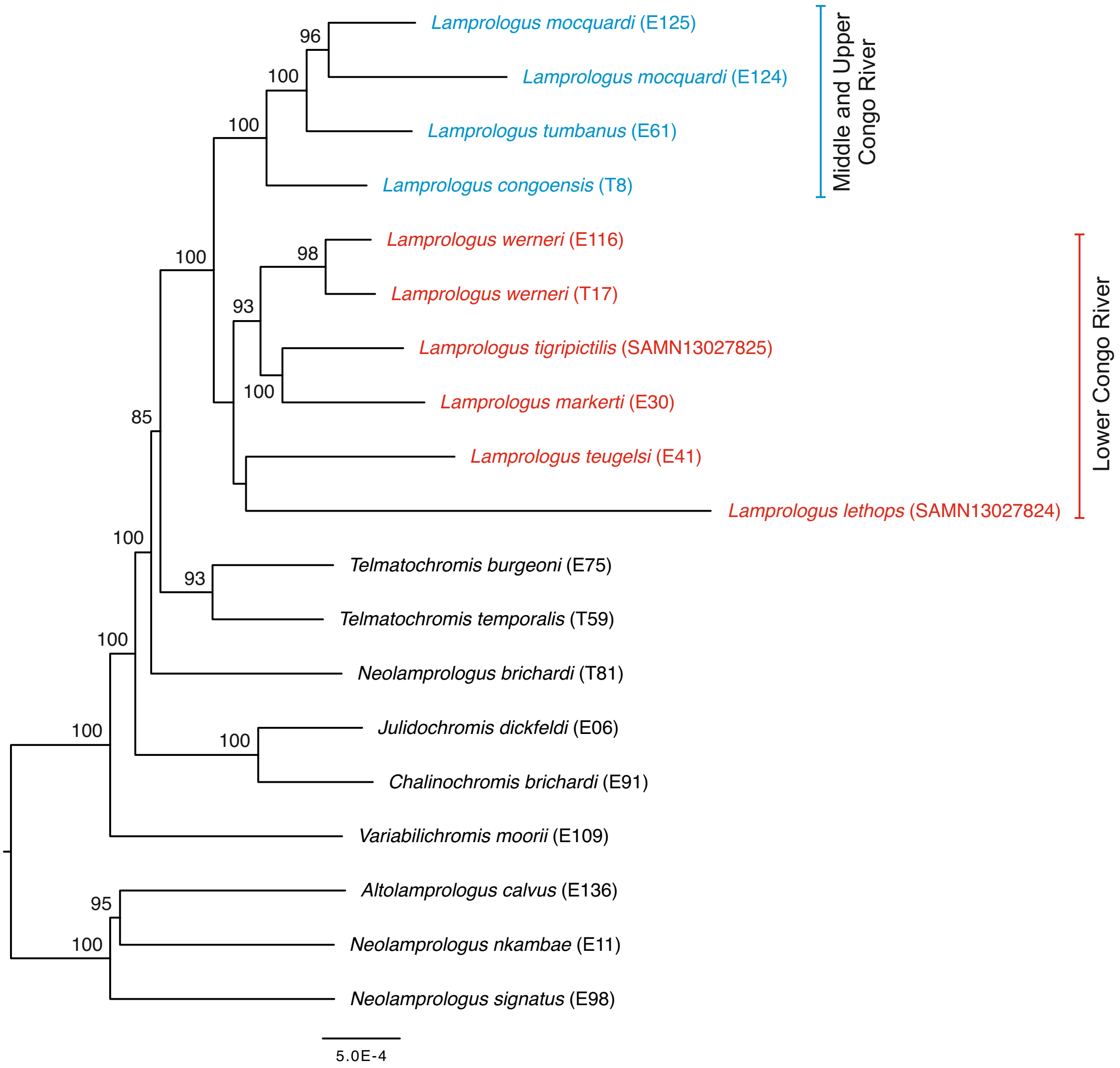

Fig. S2 Maximum Likelihood (IQ-TREE2) phylogeny based on the concatenated analysis of ultraconserved elements (409 loci and 181,498 bp) of riverine *Lamprologus* and related lamprologines analyzed in this study and in Alda et al. 2025. Species endemic to the Lower Congo River are marked in red, and those endemic to the Middle and Upper Congo River are shown in blue. Node labels indicate bootstrap values.

Table S1. Characteristics of the mitochondrial genomes of the Lamprologus species analyzed in this study. The first and last nucleotide positions (bp) are shown for L. lethops and the differences in size relative to this specimen are shown for the rest of species (rel. size). The start and stop codons are indicated for each coding gene.

	L. lethops		*L. congoensis* T12		*L. congoensis* T8		*L. markerti* E30		L. mocquardi E124		*L. mocquardi* E125		*L. teugelsi* E41		*L. tigripictilis*		*L. tumbanus* E61		*L. werneri* E116		*L. werneri* T17	
Amino acid/gene	bp	start/stop	rel. size	start/stop	rel. size	start/stop	rel. size	start/stop	rel. size	start/stop	rel. size	start/stop	rel. size	start/stop	rel. size	start/stop	rel. size	start/stop	rel. size	start/stop	rel. size	start/stop
tRNA-Phe	1-69		0		0		0		0		0		0		0		0		0		0	
12S rRNA	70-1013		2		2		0		2		2		0		0		1		2		2	
tRNA-Val	1014-1085		0		0		0		0		0		0		0		0		0		0	
16S rRNA	1086-2773		-2		-4		1		-3		-4		-1		0		-4		-3		-5	
tRNA-Leu	2774-2847		0		0		0		0		0		0		0		0		0		0	
ND1	2848-3822	ATG/TAG	0	ATG/TAG	0	ATG/TAG	0	ATG/TAG	0	ATG/TAG	0	ATG/TAG	0	ATG/TAA	0	ATG/TAG	0	ATG/TAG	0	ATG/TAG	0	ATG/TAG
tRNA-Ile	3826-3895		0		0		0		0		0		0		0		0		0		0	
tRNA-Gln	3965-3895		0		0		0		0		0		0		0		0		0		0	
tRNA-Met	3965-4033		0		0		0		0		0		0		0		0		0		0	
ND2	4034-5078	ATG/T--	-1	ATG/TA-	-1	ATG/TA-	0	ATG/T--	-1	ATG/TA-	-1	ATG/TA-	0	ATG/T--	0	ATG/T--	-1	ATG/TA-	-1	ATG/TA-	-1	ATG/TA-
tRNA-Trp	5079-5150		0		0		0		0		0		0		0		0		0		0	
tRNA-Ala	5220-5152		0		0		0		0		0		0		0		0		0		0	
tRNA-Asn	5294-5222		0		0		0		0		0		0		0		0		0		0	
tRNA-Cys	5395-5330		1		0		0		1		0		0		0		0		0		0	
tRNA-Tyr	5465-5396		0		0		0		0		0		0		0		0		0		0	
COX1	5467-7062	GTG/TAA	0	GTG/TAA	0	GTG/TAA	0	GTG/TAA	0	GTG/TAA	0	GTG/TAA	0	GTG/TAA	0	GTG/TAA	0	GTG/TAA	0	GTG/TAA	0	GTG/TAA
tRNA-Ser	7133-7063		0		0		0		0		0		0		0							
tRNA-Asp	7137-7209		0		0		0		0		0		0		0		0		0		0	
COX2	7215-7905	ATG/T--	0	ATG/T--	0	ATG/T--	0	ATG/T--	0	ATG/T--	0	ATG/T--	0	ATG/T--	0	ATG/T--	0	ATG/T--	0	ATG/T--	0	ATG/T--
tRNA-Lys	7906-7979		0		0		0		0		0		0		0		0		0		0	
ATP8	7981-8148	ATG/TAA	0	ATG/TAA	0	ATG/TAA	0	ATG/TAA	0	ATG/TAA	0	ATG/TAA	0	ATG/TAA	0	ATG/TAA	0	ATG/TAA	0	ATG/TAA	0	ATG/TAA
ATP6	8139-8821	ATG/TAA	0	ATG/TAA	0	ATG/TAA	0	ATG/TAA	0	ATG/TAA	0	ATG/TAA	0	ATG/TAA	0	ATG/TAA	0	ATG/TAA	0	ATG/TAA	0	ATG/TAA
COX3	8822-9605	ATG/T--	0	ATG/T--	0	ATG/T--	0	ATG/T--	0	ATG/T--	0	ATG/T--	0	ATG/T--	0	ATG/T--	0	ATG/T--	0	ATG/T--	0	ATG/T--
tRNA-Gly	9606-9677		0		0		0		0		0		0		0		0		0		0	
ND3	9678-10026	ATG/T--	0	ATG/T--	0	ATG/T--	0	ATG/T--	0	ATG/T--	0	ATG/T--	0	ATG/T--	0	ATG/T--	0	ATG/T--	0	ATG/T--	0	ATG/T--

tRNA-Arg	10027-10095		0		0		0		0		0		0		0		-1		0		0	
ND4L	10096-10392	ATG/TAA	0	ATG/TAA	0	ATG/TAA	0	ATG/TAA	0	ATG/TAA	0	ATG/TAA	0	ATG/TAA	0	ATG/TAA	0	ATG/TAA	0	ATG/TAA	0	ATG/TAA
ND4	10386-11766	ATG/T--	0	ATG/T--	0	ATG/T--	0	ATG/T--	0	ATG/T--	0	ATG/T--	0	ATG/T--	0	ATG/T--	0	ATG/T--	0	ATG/T--	0	ATG/T--
tRNA-His	11767-11835		0		0		0		0		-1		0		0		0		0		0	
tRNA-Ser	11836-11902		0		0		0		0		0		0		0		0		0		0	
tRNA-Leu	11907-11979		0		0		0		0		0		0		0		0		0		0	
ND5	11980-13818	ATG/TAA	0	ATG/TAA	0	ATG/TAA	0	ATG/TAA	0	ATG/TAA	0	ATG/TAA	0	ATG/TAA	0	ATG/TAA	0	ATG/TAA	0	ATG/TAA	0	ATG/TAA
ND6	14336-13815	ATG/TAA	0	ATG/TAA	0	ATG/TAA	0	ATG/TAA	0	ATG/TAA	0	ATG/TAA	0	ATG/TAA	0	ATG/TAA	0	ATG/TAA	0	ATG/TAA	0	ATG/TAA
tRNA-Glu	14405-14337		0		0		0		0		0		0		0		0		0		0	
CYTB	14410-15550	ATG/T--	0	ATG/T--	0	ATG/T--	0	ATG/T--	0	ATG/T--	0	ATG/T--	0	ATG/T--	0	ATG/T--	0	ATG/T--	0	ATG/T--	0	ATG/T--
tRNA-Thr	15551-15622		0		0		0		0		0		0		0		0		0		0	
tRNA-Pro	15692-15623		0		0		0		0		0		0		0		0		0		0	
control region	15693-16578		-3		-2		-4		-1		-3		-2		-3		-1		-1		-2	
